# Guanidinoacetic acid in laying hen diets with varying dietary energy: Productivity, antioxidant status, yolk fatty acid profile, hepatic lipid metabolism, and gut health

**DOI:** 10.1016/j.psj.2025.105159

**Published:** 2025-04-13

**Authors:** Hossein Ali Ghasemi, Mohammad Azizollahi, Mahdi Ajoudani Lahroudi, Kamran Taherpour, Iman Hajkhodadadi, Hossein Akhavan-Salamat, Ali Afsar, Mahdi Khodaei-Motlagh, Enayat Rahmatnejad

**Affiliations:** a Department of Animal Science, Faculty of Agriculture and Environment, Arak University, 38156-8-8349 Arak, Iran; b Department of Animal Science, Faculty of Agriculture, Ilam University, Ilam, Iran; c Department of Animal Science, Faculty of Agriculture, Khoy Branch, Islamic Azad University, Khoy, Iran; d Evonik Iran PJS, 1436935313 Tehran, Iran; e Department of Animal Science, Faculty of Agriculture and Natural Resources, Persian Gulf University, Bushehr, 75169, Iran

**Keywords:** Dietary energy, Guanidinoacetate, Layer performance, Lipid metabolism, Nutrient digestibility

## Abstract

This study aimed to investigate the effects of GAA supplementation in diets differing in ME levels on productive performance, egg quality, blood parameters, yolk fatty acid profiles, hepatic expression of genes related to lipid metabolism, gut morphology, and nutrient digestibility in laying hens during their post-peak production phase. Over a 12-week period (52-64 weeks of age), 288 laying hens were randomly assigned to 6 treatments. Each treatment consisted of 8 replicates, with 6 hens per replicate. The experimental treatments were assigned in a 2 × 3 factorial arrangement, comprising 2 levels of dietary ME (a recommended level and a low level, the latter characterized by a 100 kcal/kg reduction in ME) and 3 levels of GAA supplementation (0, 0.6, and 1.2 g/kg). The results showed significant interaction effects (*P* < 0.05) between GAA supplementation and dietary ME levels on laying rate, egg mass, feed conversion ratio, crude protein digestibility, and AMEn. In hens fed the low-ME diet, GAA supplementation, particularly at 1.2 g/kg, significantly improved laying performance. Moreover, at both 0.6 and 1.2 g/kg under low-ME conditions, GAA significantly enhanced crude protein digestibility and AMEn. The low-ME diet was associated with decreased expression of key lipogenic genes, including sterol regulatory element-binding transcription factor 1 (SREBF1), acetyl-coenzyme A carboxylase (ACC), and fatty acid synthase (FAS), alongside increased expression of genes involved in fatty acid oxidation, such as peroxisome proliferator-activated receptor alpha (PPARα) and carnitine palmitoyltransferase 1 (CPT1). Regardless of ME content, GAA supplementation linearly improved eggshell strength, enhanced the polyunsaturated-to-saturated fatty acid ratio in the yolk, elevated serum levels of creatine and total antioxidant capacity, improved intestinal morphology, and increased radical scavenging activity in the yolk (*P* < 0.05). Furthermore, GAA supplementation linearly increased the relative mRNA expression of several metabolic genes, including SREBF1, ACC, PPARα, and ApoB (*P* < 0.05). In conclusion, GAA supplementation enhanced productive performance in low-ME diets and exerted positive effects on egg characteristics and lipid metabolism, regardless of dietary ME content.

## Introduction

During the post-peak period of lay, declines in egg production and shell quality are common, often leading to considerable economic losses. Sustaining productivity during this stage requires careful nutritional management to support the recovery of body reserves and continued egg production (Junqueira et al., 2006; Liu et al., 2013). Optimizing dietary metabolizable energy (ME) is particularly important, as energy demands remain high while energy-dense feed ingredients are costly (dePersio et al., 2015). A reduction of 100 kcal during the post-peak production phase in laying hens can significantly impair egg production, resulting in decreased egg size and quality due to inadequate energy availability for efficient biosynthesis (Mikulski et al., 2020). This energy deficit may trigger the mobilization of body fat reserves, potentially altering body composition and reducing feed conversion efficiency (Barzegar et al., 2019). Although laying hens are generally capable of adapting to moderate energy restrictions, they often maintain egg production rates above 85 % when the deficit is not severe (Leeson and Summers, 2008), optimizing the utilization of dietary metabolizable energy (ME) remains a key objective in poultry nutrition, particularly in the context of low-ME dietary formulations. One promising strategy involves enhancing intracellular energy availability by improving the storage and transfer of energy through phosphate bond formation, thereby increasing the efficiency of cellular energy metabolism (Mousavi et al., 2013; Ale Saheb Fosoul et al., 2018).

Guanidinoacetic acid (GAA), also known as guanidinoacetate or glycocyamine, is a feed additive that enhances energy metabolism by promoting creatine biosynthesis (Khajali et al., 2020). In birds, endogenous creatine synthesis is limited, and a portion of it is irreversibly converted to creatinine and excreted, increasing reliance on dietary sources (Asiriwardhana and Bertolo, 2022). Due to the instability and cost of creatine, GAA offers a more practical alternative (Baker, 2009). Previous studies have shown that GAA supplementation improves productivity and egg characteristics in laying hens (Salah et al., 2020; Azizollahi et al., 2024). GAA may also exert arginine-sparing effects, enhance nutrient digestibility (Portocarero and Braun, 2021), and strengthen antioxidant defenses (Zhao et al., 2021; Amiri et al., 2019). Assessing its effects under energy-deficient conditions is important to evaluate its potential as a compensatory nutritional strategy. As a direct precursor to creatine, GAA supports ATP regeneration and has shown beneficial effects in low-energy diets (Mousavi et al., 2013; Ceylan et al., 2021), which may be particularly advantageous during the post-peak production phase, when the metabolic demands of laying hens remain high.

Lipid metabolism in animals is a complex process regulated by numerous genes. Genes such as sterol regulatory element-binding transcription factor 1 (SREBF1), acetyl-coenzyme A carboxylase (ACC), carnitine palmitoyltransferase 1 (CPT1), peroxisome proliferator-activated receptor alpha (PPARα), fatty acid synthase (FAS), and lipoprotein lipase (LPL) play key roles in lipid synthesis and degradation. Apolipoprotein B (ApoB) is also crucial for lipid transport and deposition (Kirchgessner et al., 1987; Hu et al., 2008; Ge et al., 2019). GAA has been found to stimulate phosphocreatine synthesis, thereby supplying energy to metabolically active tissues such as muscle, brain, and gonads. This process supports improved nutrient distribution and enhances animal productivity (Michiels et al., 2012). Exogenous GAA supplementation may also regulate energy partitioning across tissues (Cui et al., 2022). Previous studies in pigs (Lu et al., 2020) and ducks (Wu et al., 2024) have shown that GAA can inhibit hepatic fatty acid synthesis and promote fatty acid β-oxidation by modulating the activity of key metabolic enzymes. However, limited research has investigated the effects of GAA on lipid metabolism and fat utilization in laying hens, which have elevated metabolic demands due to egg production. Therefore, examining the impact of GAA supplementation in layer diets may help optimize the nutritional profile of eggs and offer implications for human health.

Based on this background, the interaction between GAA supplementation and dietary energy levels in aged laying hens is of particular importance for optimizing performance. It is hypothesized that GAA's effects on energy utilization and nutrient digestibility may vary depending on dietary ME levels, thereby influencing lipid metabolism and yolk fatty acid composition. Accordingly, the objective of this study was to evaluate the effects of different GAA supplementation levels under varying dietary ME conditions on productive performance, egg quality traits, blood biochemical indices, intestinal morphology, yolk fatty acid composition, hepatic lipid metabolism, and nutrient digestibility in laying hens.

## Materials and methods

### Diets and experimental design

The welfare of the birds and the experimental procedures employed in this study were approved by the Animal Care and Use Committee at Arak University (contract number 1401-8687) and followed the welfare and care instructions for birds as outlined in the ARRIVE guidelines. The study involved 288 Super Nick white laying hens selected from a commercial flock. All hens were 52 weeks old and exhibited uniform body weight and productivity. The study was conducted over a 12-week period, employing a 2 × 3 factorial arrangement based on a completely randomized design. A total of 6 dietary treatments were applied, with each treatment replicated in 8 cages containing 6 laying hens per cage. The dietary treatments included 2 levels of ME (a recommended or normal level, and a reduced level with a 100 kcal/kg reduction in ME) and 3 concentrations of GAA (0, 0.6, or 1.2 g/kg of diet). Birds were kept in cages with a stocking density of 675 cm^2^ per hen and were provided with linear feeders and nipple drinkers. The ambient temperature was maintained at a minimum of 20°C, and lighting was provided 16 h per day. The GAA supplementation was supplied as GuanAMINO® (Evonik Operations GmbH, Germany), with a purity exceeding 96 %. Experimental diets were formulated in mash form according to the nutritional recommendations outlined in the Super Nick Nutrition Management Guide. The ingredient composition and nutrient contents of the diets are presented in Table 1, Table 2.

**Table 1:** Composition of normal- and low-metabolizable energy (ME) diets (as fed basis), fed to laying hens (weeks 52 to 64).

Ingredients (g/kg)	Normal ME	Low ME
Corn	601.2	572.3
Soybean meal	262.2	253.8
Soybean oil	8.5	3.9
Wheat bran	-	42.2
Oyster shell	50.0	50.0
Limestone	52.5	52.7
Dicalcium phosphate	14.8	14.3
Salt (NaCl)	2.0	2.3
Sodium bicarbonate	1.0	0.6
Vitamin and mineral premix^1^	5.0	5.0
DL-Methionine	1.8	1.9
L-Lysine HCl	0.6	0.6
L-Threonine	0.4	0.4

1 Supplied per kg of diet: all-trans-retinyl acetate, 8800 IU; cholecalciferol, 2500 IU; α-tocopherol acetate, 6.6 mg; menadione sodium bisulfite, 2.5 mg; thiamine mononitrate, 1.5 mg; riboflavin, 4.4 mg; nicotinic acid, 20 mg; calcium d-pantothenate, 8 mg; pyridoxine, 2.5 mg; folic acid, 1.1 mg; cyanocobalamine, 0.08 mg; biotin, 0.15 mg; choline chloride, 400 mg, Mn (from manganese sulphate), 60 mg; Fe (from ferrous sulphate), 30 mg; Zn (from zinc sulphate), 66 mg; Cu (from copper sulphate), 6 mg; I (from potassium iodate), 0.8 mg; Se (from sodium selenite), 0.2 mg.

**Table 2:** calculated and determined analysis of diets (as-fed basis), fed to laying hens (weeks 52 to 64).

	Normal ME	Low ME
Calculated nutritive value (g/kg unless stated otherwise)		
Metabolizable energy, kcal/kg	2,700	2,600
Crude protein	16.7	16.7
Calcium	43.0	43.0
Nonphytate phosphorous	3.8	3.8
Digestible TSAA^1^	6.7	6.7
Digestible lysine	7.5	7.5
Digestible threonine	5.2	5.2
Sodium	1.6	1.6
DEB^2^, mEq/kg	230	230
Analyzed nutritive value^3^ (g/kg)		
Total Energy, kcal/kg	3,475	3,392
Crude protein	172.9	174.2
Ether extract	34.6	30.2
Crude fiber	30.3	33.0
Total ash	139.2	140.5
Total phosphorous	5.6	5.8
Calcium	40.5	40.5
Amino acid profile		
Total lysine	9.6	9.5
Total methionine	4.7	4.7
Total cysteine	3.0	3.0
TSAA	7.9	7.9
Total threonine	7.1	7.0
Total valine	9.2	9.2
Total tryptophan	2.2	2.2
Total arginine	10.2	10.2
Fatty acid profile (% of total fatty acids)		
C16:0	9.34	9.74
C18:0	2.11	2.04
C18:1n-9	21.94	21.63
C18:2n-6	50.39	50.14
C18:3n-3	2.73	2.85
PUFA/SFA ratio	4.64	4.50

1 Total sulfur amino acids.

2 DEB (dietary electrolyte balance) = (Na+, mEq/kg + *K*+, mEq/kg) – CL−, mEq/kg.

3 Mean of three samples per diet.

### Chemical analysis

The gross energy and nutritional composition (dry matter, protein, fat, ash, fiber, calcium, and phosphorus) of corn, soybean meal, wheat bran, and the experimental diets, were determined using standardized methods (AOAC, 2006). The amino acid content of feed ingredients and diets was also analyzed, and digestibility coefficients were calculated using AMINODat 5.0 (Evonik Operations GmbH, 2015).

### Productive performance

Feed intake was recorded weekly on a per-cage basis. To determine the average daily feed intake per bird, the total feed consumed during the 12-week feeding period (from 52 to 64 weeks of age) was divided by the number of bird-days. Egg production was monitored daily, and both the number and weight of eggs were recorded. Average egg weight and daily egg production were used to calculate egg mass. The feed conversion ratio (FCR) was determined by dividing total feed intake by egg mass. Additionally, final body weights of the hens were recorded at the end of the study.

### Sample collection and measurements

At the conclusion of the experiment (64 weeks of age), 16 birds per treatment group (2 birds per replicate cage) were randomly selected for sample collection. These birds were sedated via intramuscular injection of ketamine hydrochloride (100 mg) and euthanized through cervical dislocation. Liver samples were collected for gene expression analysis related to lipid metabolism, preserved in liquid nitrogen, and stored at −80 °C for later analysis. Ileal contents were also collected by pooling samples from each replicate cage and stored at −20 °C for digestibility assessments. The remaining birds were euthanized using a carbon dioxide chamber at the end of the study.

For egg quality assessment, 4 eggs per replicate cage were collected at 64 weeks of age, and measurements were performed within 24 h after deposition. Yolks from each replicate (*n* = 8 per treatment) were separated, pooled, homogenized, and stored at −20 °C for further analysis. To evaluate lipid oxidation in egg yolks, additional samples were collected from 32 eggs per treatment, resulting in 8 pooled yolk samples per treatment group.

### Egg Quality Indicators

Eggshell strength was measured using an eggshell strength tester (OSK 13473, Japan). Following this assessment, albumen and yolk components were manually separated. A digital scale with a precision of 0.01 g was used to weigh the albumen and yolk. Yolk color was evaluated using the Roche color fan scale, ranging from 15 (dark orange) to 1 (light pale yellow). A digital caliper with a precision of 0.01 mm was used to measure albumen height. The albumen height and egg weight were then used to calculate the Haugh unit, as described by Eisen et al. (1962). The dry weight of the eggs was recorded, and eggshell thickness was measured using a digital caliper (±0.01 mm). Three measurements were taken at different points on each shell, and the mean value was reported as the average eggshell thickness.

### Serum parameters

At 64 weeks of age, blood samples were collected from 2 hens per replicate (16 hens per treatment group). Samples were centrifuged at 3,000 × *g* for 10 min at 4 °C to isolate the serum, which was subsequently stored at −80 °C until analysis. Serum concentrations of cholesterol, triglycerides, total protein, albumin, glucose, and uric acid were measured using commercial biochemical kits (Pars Azmoun Co., Tehran, Iran) and an automated spectrophotometer (Chem 200, Gesan Production Srl, Campobello, Italy). Levels of creatine and creatinine were quantified using spectrophotometric kits (Sigma-Aldrich Company Ltd., Poole, Dorset, UK). Malondialdehyde (MDA) levels were assessed via reaction with 2-thiobarbituric acid, with absorbance measured at 532 nm (Jensen et al., 1997). Total antioxidant capacity (TAC) of the serum was evaluated using the ABTS assay and Randox kits (Pars Azmoun Co., Tehran, Iran). Serum nitric oxide (NO) concentrations were determined using the Griess reagent, with absorbance measured at 540 nm (Haddad et al., 1995).

### Assessment of lipid oxidation and antioxidant capacity in egg yolk

To assess lipid oxidation in egg yolk, samples were collected from 32 eggs, resulting in 8 pooled samples per treatment. The analysis employed the 2-thiobarbituric acid reactive substances (TBARS) method. Malondialdehyde (MDA) levels were quantified using a standard curve generated from tetraethoxypropane hydrolysis (T9889, 97 %, Sigma, USA). Absorbance was measured at 521.5 nm, and results were expressed as mg MDA per kg of fresh yolk.

For the DPPH radical scavenging assay, 8 pooled yolk samples per treatment (from 32 fresh egg yolks) were extracted. DPPH activity was evaluated following the procedure of Zhang et al. (2014). Briefly, 400 μL of each sample was mixed with 3.6 mL of 0.1 mM methanolic DPPH solution, incubated in the dark for 30 min, and the absorbance was measured at 517 nm using a UV-Vis spectrophotometer (Model UV-2550, Shimadzu, Japan). Radical scavenging activity (%) was calculated by comparing the sample absorbance to that of the control.

### Fatty acid profile measurement

Pooled yolk samples (*n* = 8 per treatment) were homogenized and stored at −20 °C until analysis. Fatty acid separation and quantification were performed using gas chromatography (Model 4600, Unicam, Cambridge, UK), following the procedure described by Ghasemi et al. (2022). Fatty acids were identified and quantified based on retention times and peak areas, using C15:0 (Sigma, St. Louis, MO, USA) as the internal standard. Results were expressed as percentages of total fatty acids. The sums of saturated (SFA), monounsaturated (MUFA), and polyunsaturated fatty acids (PUFA) were subsequently calculated.

### RNA extraction and gene expression analysis

Total RNA was extracted from liver samples using a commercial RNA extraction kit. Initially, frozen liver samples were ground in liquid nitrogen, followed by the addition of RNA extraction solution to lyse the cells. After vortexing, 200 μL of chloroform was added, and the samples were centrifuged to isolate the RNA-containing upper phase. RNA was precipitated with 200 μL of isopropanol, collected via centrifugation at 12,000 × *g* for 10 min at 4°C, washed with 1 mL of ethanol, dried, and resuspended in DEPC-treated water. RNA concentration and purity were determined using a microplate spectrophotometer (BioTek, USA).

Genomic DNA was removed using DNase I, and complementary DNA (cDNA) was synthesized with a commercial cDNA synthesis kit (SinaClon, Tehran, Iran). Each qPCR reaction was prepared using 25 ng of cDNA template, forward and reverse primers, SYBR Green master mix, and DEPC-treated water. Relative expression levels of genes involved in lipid metabolism, including ACC, FAS, LPL, SREBF1, PPARα, CPT1, and ApoB, were quantified by real-time PCR using β-ACTIN as the reference gene. Primer sequences are provided in Table 3. Reactions were run under uniform thermal cycling conditions, and gene expression levels were calculated using the 2^−ΔΔCt^ method (Livak and Schmittgen, 2001). Expression values were normalized to the control group.

**Table 3:** Gene special primers used for quantitative real-time PCR.

Gene	Primer sequence 5′-3′	Length (nt)	GenBank number
ACC	F: TGTGGCTGATGTGAGCTTTC	152	NM_205505.1
	R: ACTGTCGGGTCACCTTCAAC		
FAS	F: TTGGCTGTGAGTGGGAAGAT	206	NM_205155.2
	R: GCACTTCCTTCACTGTTGCA		
LPL	F: CAGTGCAACTTCAACCATACCA	150	NM_205282.2
	R: AACCAGCCAGTCCACAACAA		
SREBF1	F: AGACCTCCAGCATCACCTCT	234	NM_204126.2
	R: TGTCGTTGATGGATGAGCGG		
CPT1	F: TCGTCTTGCCATGACTGGTG	143	NM_001012898.1
	R: GCTGTGGTGTCTGACTCGTT		
PPARα	F: TGTGGAGATCGTCCTGGTCT	103	NM_001001464
	R: CGTCAGGATGGTTGGTTTGC		
ApoB	F: AGGTGGTGGTGAAGAGGTGGAGAG	97	NM_001044633.1
	R: GAGCAGCAAGAGCCGCACAG		
β-ACTIN	F: TTACTCGCCTCTGTGAAGGC	228	NM_205518.1
	R: TCCTAGACTGTGGGGGACTG		

*F*, forward primer; *R*, reverse primer; ACC, acetyl-CoA carboxylase alpha; FAS, fatty acid synthase; LPL, lipoprotein lipase; SREBF1, sterol regulatory element binding transcription factor 1; CPT1, carnitine palmitoyl transferase 1; PPARα, peroxisome proliferator activated receptor alpha; ApoB, apolipoprotein B.

### Gut morphology

Intestinal samples were processed through dehydration, clearing, and paraffin embedding. Cross-sections of 5 μm thickness were prepared using a microtome (Typ1400, Leitz, Wetzlar, Germany), as described by Amiri et al. (2019). These sections were mounted on glass slides and stained with hematoxylin and eosin following standard histological protocols. The stained slides were examined under a light microscope (Olympus CX31, Shinjuku, Tokyo, Japan). Morphological parameters, including villus height (VH) and crypt depth (CD), were measured using image analysis software (QWinPlus version 3.1.0, Leica Cambridge Ltd., Cambridge, UK). For each bird, 2 intestinal sections were evaluated, with a minimum of 10 villi and 10 crypts measured per section.

### Nutrient digestibility

The apparent ileal digestibility (AID) of nutrients was determined using acid-insoluble ash (AIA) as an indigestible marker. Celite, a source of AIA, was added to all diets at a concentration of 5 g/kg during the final 4 days of the experimental period to ensure accurate digestibility estimation (Abbasi Arabshahi et al., 2021). Ileal digesta were freeze-dried and ground using a hammer mill fitted with a 0.5-mm screen. Chemical analyses were performed on the digesta samples using the same procedures employed for feed and diet analysis. AIA concentrations in both feed and digesta were determined following the method of McCarthy et al. (1974). AID (%) was calculated using the following equation:

$$\text{AID}\left(\%\right)=\left[1-\left(\text{AI}A_{\text{feed}}/\text{AI}A_{\text{id}}\right)\times \left(\text{nutrien}t_{\text{id}}/\text{nutrien}t_{\text{feed}}\right)\right]\times 100$$

Where AIA _feed_ and nutrient _feed_ are the concentrations of AIA and nutrient in the feed (%) and AIA _id_ and nutrient _id_ represent the concentrations (%) of the AIA and nutrient in the ileal digesta.

The AMEn was determined from excreta samples (*n* = 8 per treatment) using the following formula:

AMEn was determined using the same procedure described for nutrient digestibility, with AIA serving as an internal marker. Excreta were collected from 8 cages per treatment (*n* = 8) over a 72-hour period, pooled per cage, dried at 65 °C, and ground to a uniform particle size. Gross energy and nitrogen contents of the feed and excreta were analyzed using the methods previously described. AMEn was calculated using the following equation:

$$\text{AMEn}\left(\text{kcal}/\text{kg}\;\text{of}\;\text{feed}\right)=GE_{\text{feed}}--\left[\left(GE_{\text{ex}}\times \text{IF}\right)+8.22\times \left(N_{\text{feed}}--N_{\text{ex}}\times \text{IF}\right)\right]$$

In this equation, GE _feed_ and GE _ex_, respectively, represent the gross energy content (kcal/kg) in the diet and excreta; IF is the indigestibility factor calculated as AIA _feed_/AIA _ex_; N _feed_ is nitrogen content in diet (%); N _ex_ is nitrogen content in excreta (%); and 8.22 is the energy equivalent (kcal/g) of uric acid.

### Statistical analysis

All data were analyzed using SAS software (version 9.0; SAS Institute, Cary, NC, USA) via a two-way analysis of variance (ANOVA) to assess the main effects of dietary ME level and GAA concentration, as well as their interaction in a 2 × 3 factorial design. Orthogonal polynomial contrasts were used to evaluate linear and quadratic responses to increasing dietary GAA levels. The assessment of yolk color was performed using a one-way non-parametric test (the NPAR1WAY procedure and Kruskal-Wallis test) because a hedonic scale was used. The experimental unit depended on the measured variable: the cage served as the unit for productive performance and egg quality parameters, while individual birds were used for all other analyses. Normality and homogeneity of variance were assessed using the Shapiro–Wilk test and Levene's test, respectively. Differences among treatment means were considered statistically significant at *P* < 0.05 and were further evaluated using Tukey's post hoc test. Data are reported as means ± standard error of the mean (SEM).

## Results

### Productive performance

Table 4 presents the effects of dietary ME content and GAA supplementation on performance indicators in laying hens. A significant interaction between dietary ME and GAA was observed for laying rate, egg mass, and FCR (*P* < 0.05). In the low-ME group, GAA at 0.6 and 1.2 g/kg significantly increased laying rate (85.22 % to 86.65 % and 88.96 %) and egg mass (55.27 to 56.66 and 58.30 g/hen/day), and reduced FCR (1.99 to 1.92 and 1.84 g/g). No significant changes occurred in the normal-ME group. Regarding main effects, a reduction of 100 kcal/kg in dietary ME resulted in significantly increased feed intake and FCR (*P* < 0.05), without affecting egg weight, production rate, egg mass, or final body weight (*P* > 0.05). GAA supplementation significantly enhanced laying rate and egg mass, and reduced FCR (linear trend; *P* < 0.001). However, it had no significant effects on feed intake, egg weight, or final body weight (*P* > 0.05).

**Table 4:** Productive performance (from 52 to 64 weeks of age) according to the metabolizable energy (ME) content and guanidinoacetic acid (GAA) supplementation in the diets of the laying hens.

ME level	GAA level (g/kg diet)	Laying rate	Egg weight	Egg mass	Feed consumption	Feed conversion ratio	Body weight
		%	g	g/hen/d	g/hen/d	g/g	g
Normal	0	86.96^bc^	65.42	56.89^b^	106.8	1.88^bc^	1,715
Normal	0.6	87.57^bc^	65.23	57.12^ab^	106.9	1.87^bc^	1,732
Normal	1.2	88.21^ab^	65.27	57.58^ab^	106.7	1.85^c^	1,697
Low	0	85.22^d^	64.85	55.27^c^	109.9	1.99^a^	1,680
Low	0.6	86.65^c^	65.39	56.66^b^	108.7	1.92^b^	1,696
Low	1.2	88.96^a^	65.53	58.30^a^	107.5	1.84^c^	1,672
SEM		0.464	0.359	0.421	0.69	0.021	23.0
Main effect							
ME		87.56	65.30	57.19	106.8^b^	1.87^b^	1,715
Normal		86.94	65.26	56.74	108.7^a^	1.92^a^	1,683
Low		0.268	0.207	0.243	0.40	0.012	13.3
SEM							
GAA level (g/kg diet)							
0		86.09^c^	65.14	56.08^b^	108.3	1.93^a^	1,698
0.6		87.11^b^	65.31	56.89^b^	107.8	1.90^a^	1,714
1.2		88.59^a^	65.40	57.94^a^	107.1	1.85^b^	1,684
SEM		0.328	0.254	0.298	0.49	0.014	16.2
*P*-value							
ME level		0.101	0.873	0.197	0.001	0.006	0.096
GAA level		<0.001	0.755	<0.001	0.197	<0.001	0.444
ME × GAA		0.032	0.462	0.029	0.245	0.026	0.964
Effect of GAA level							
Linear		<0.001	0.464	<0.001	0.074	<0.001	0.564
Quadratic		0.573	0.889	0.748	0.879	0.903	0.258

Means within each column series with no superscript letters are not significantly different (*P* > 0.05). Values are means of 8 replicates per treatment combination with 6 laying hens per replicate.

### Egg quality

As shown in Table 5, dietary ME content and its interaction with GAA supplementation did not significantly affect yolk, albumen, shell weights, yolk color, albumen height, Haugh unit, or shell quality indices (*P* > 0.05). However, GAA supplementation led to a linear increase in Haugh unit (*P* < 0.05) and showed trends toward higher eggshell relative weight (*P* = 0.082) and eggshell strength (*P* = 0.073), though other qualitative parameters remained unaffected (*P* > 0.05).

**Table 5:** Egg quality parameters (at 64 weeks of age) according to the metabolizable energy (ME) content and guanidinoacetic acid (GAA) supplementation in the diets of the laying hens.

		ME level			GAA level (g/kg diet)				*P*-value		
Item		Normal	Low	SEM	0	0.6	1.2	SEM	ME level	GAA level	ME × GAA
% of egg components	Yolk	28.73	28.61	0.264	28.76	28.79	28.46	0.323	0.767	0.728	0.947
	Albumen	61.72	61.68	0.324	61.94	61.45	61.70	0.397	0.942	0.684	0.934
	Shell	9.56	9.70	0.145	9.30	9.75	9.84	0.178	0.488	0.082	0.957
											
Albumin height	mm	5.97	5.86	0.138	5.64	5.91	6.18	0.17	0.583	0.103	0.994
Haugh Unit^1^		83.21	82.99	0.596	81.60^b^	83.29^ab^	84.41^a^	0.73	0.802	0.032	0.613
Yolk color		7.63	7.08	0.272	7.19	7.38	7.50	0.333	0.177	0.858	0.782
Shell thickness	mm	0.343	0.337	0.0045	0.333	0.339	0.349	0.0055	0.338	0.139	0.991
Shell strength	kg/cm^2^	3.30	3.25	0.055	3.17	3.27	3.39	0.068	0.476	0.073	0.971

Means within each column series with no superscript letters are not significantly different (*P* > 0.05). Values are means of 8 replicates per treatment combination with 4 eggs per replicate.

1 There was a significant linear effects of dietary GAA levels (*P* < 0.05).

### Serum biochemical parameters

Table 6 summarizes the effects of dietary ME and GAA on serum biochemical parameters, including metabolites, creatine, creatinine, NO, and antioxidant indices. No significant interaction effects were observed for any parameter (*P* > 0.05). Reducing dietary ME significantly increased serum uric acid levels (*P* < 0.001). Increasing dietary GAA resulted in a linear increase in creatine, creatinine, and NO concentrations (*P* < 0.001), with the 1.2 g/kg GAA group displaying the highest values for creatine and NO. Additionally, GAA supplementation significantly increased TAC (linear and quadratic trends; *P* < 0.05) and reduced MDA concentration (linear and quadratic trends; *P* < 0.05). The highest TAC and lowest MDA values were observed with a dietary GAA level of 0.6 g/kg.

**Table 6:** Serum parameters (at 64 weeks of age) according to metabolizable energy (ME) content and guanidinoacetic acid (GAA) supplementation in the diets of the laying hens.

		ME level			GAA level (g/kg diet)				*P*-value		
Item		Normal	Low	SEM	0	0.6	1.2	SEM	ME level	GAA level	ME × GAA
Glucose	mg/dL	251	237.1	8.78	233.1	249.7	249.3	10.75	0.269	0.468	0.824
Cholesterol	mg/dL	140.6	132.5	5.21	143.5	131.4	134.7	6.38	0.273	0.391	0.754
Triglyceride	mg/dL	1,195	1,140	30.8	1,206	1,104	1,191	37.7	0.214	0.132	0.576
Protein	g/dL	5.12	4.9	0.128	4.86	5.01	5.17	0.157	0.238	0.385	0.967
Albumin	g/dL	2.62	2.37	0.099	2.38	2.49	2.61	0.121	0.083	0.416	0.973
Uric acid	mg/dL	5.25^b^	6.06^a^	0.148	5.80	5.63	5.53	0.181	<0.001	0.567	0.759
Creatine^1^	μmol/L	25.98	25.82	0.522	16.07^c^	25.25^b^	36.38^a^	0.640	0.827	<0.001	0.171
Creatinine^1^	µg/mL	0.459	0.484	0.0170	0.405^b^	0.442^b^	0.567^a^	0.0209	0.309	<0.001	0.942
Nitric oxide^1^	µmol/L	32.21	34.77	1.272	27.88^c^	34.03^b^	38.55^a^	1.557	0.162	<0.001	0.566
Malondialdehyde^2^	nmol/mL	2.26	2.10	0.073	2.41^a^	1.98^b^	2.15^b^	0.089	0.139	0.005	0.790
TAC^2^	mmol/L	1.54	1.53	0.048	1.35^c^	1.70^a^	1.55^b^	0.059	0.774	0.001	0.460

Means within each column series with no superscript letters are not significantly different (*P* > 0.05). Values are means of 8 replicates per treatment combination with 2 hens per replicate.

1 There was a significant linear effect of dietary GAA levels (*P* < 0.001).

2 There were significant linear (*P* < 0.05) and quadratic effects (*P* < 0.01) of dietary GAA.

### Antioxidant activity in egg yolks

**Fig 1:**
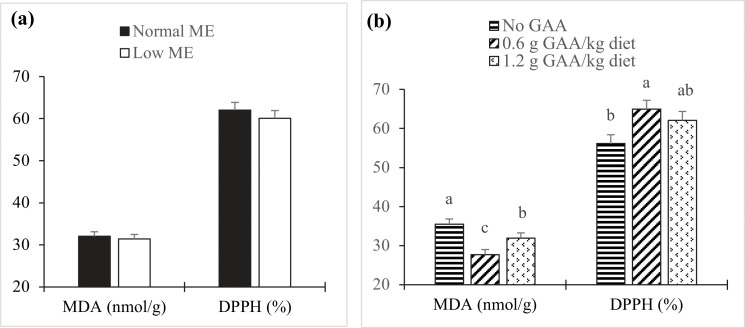
Bar charts of Lipid oxidation [(mg malondialdehyde (MDA) /kg fresh egg yolk)] and 1, 1-diphenyl-2-picrylhydrazyl (DPPH) radical scavenging activity in fresh eggs at 64 weeks of age according to the metabolizable energy (ME) content (a) and guanidinoacetic acid (GAA) supplementation (b) in the diets of laying hens. According to Tukey's test, mean values within the same histogram with no common superscript differ significantly (*P* < 0.05). Values are means of 8 replicates per treatment combination with 4 eggs per replicate. No significant interaction effects were observed between ME and GAA supplementation in terms of MDA concentration and PPH activity (*P* > 0.05). Dietary GAA levels had significant quadratic effects on MDA concentration (*P* = 0.001) and DPPH activity (*P* = 0.12).

Fig. 1 illustrates the effects of dietary treatments on lipid oxidation stability (TBARS values) and DPPH radical scavenging activity in egg yolks. Dietary ME and its interaction with GAA did not significantly influence MDA concentrations or DPPH activity in yolks (*P* > 0.05). However, increasing GAA levels led to a quadratic decrease in TBARS values and a quadratic increase in DPPH activity (*P* < 0.05). Hens receiving 0.6 g/kg GAA exhibited significantly lower MDA levels and higher DPPH scavenging activity compared to both the non-supplemented and 1.2 g/kg GAA groups (*P* < 0.05).

### Egg yolk fatty acid composition

Table 7 presents the fatty acid profile of egg yolks in response to dietary ME and GAA supplementation. No significant interaction was found between ME and GAA for any fatty acid (*P* > 0.05). A reduction in dietary ME led to lower levels of linoleic acid (C18:2n-6), total PUFA, n-6 PUFA, and the PUFA/SFA ratio (*P* < 0.05), with a trend toward higher SFA content (*P* = 0.094). GAA supplementation produced a linear decrease in palmitic acid, stearic acid, and total SFA concentrations (*P* < 0.05), while levels of total PUFA, n-6 PUFA, and the PUFA/SFA ratio increased linearly (*P* < 0.05). Other fatty acids, including n-3 PUFA, and the n-6/n-3 PUFA ratio, were not significantly affected by either factor (*P* > 0.05).

**Table 7:** chemical composition and fatty acid profile of egg yolks (at 64 weeks of age) according to the ME content and GAA supplementation in the diets of laying hens.

	ME level			GAA level (g/kg diet)				*P*-value		
Item^1^	Normal	Low	SEM	0	0.6	1.2	SEM	ME level	GAA level	ME × GAA
Chemical composition										
Dry matter	51.24	51.58	0.480	50.9	51.42	51.9	0.588	0.623	0.492	0.986
lipid	25.24	25.73	0.359	25.83	25.29	25.34	0.440	0.333	0.637	0.903
Protein	17.13	16.64	0.199	16.65	16.96	17.05	0.244	0.090	0.496	0.640
Cholesterol	14.60^a^	14.11^b^	0.137	14.64	14.22	14.20	0.168	0.017	0.135	0.457
Ash	2.93	3.08	0.081	2.83	3.06	3.13	0.099	0.173	0.095	0.761
C14:0	0.472	0.476	0.0099	0.488	0.472	0.463	0.0121	0.745	0.345	0.923
C15:0	0.058	0.06	0.0015	0.058	0.059	0.059	0.0019	0.347	0.857	0.438
C16:0^2^	25.85	26.14	0.193	26.48^a^	26.11^a^	25.40^b^	0.236	0.301	0.008	0.918
C17:0	0.124	0.128	0.0017	0.128	0.127	0.123	0.0021	0.134	0.174	0.927
C18:0^2^	7.25	7.51	0.109	7.73^a^	7.40^a^	7.00^b^	0.134	0.106	0.002	0.813
C20:0	0.069	0.067	0.0014	0.066	0.068	0.069	0.0017	0.238	0.529	0.313
C16:1	3.52	3.33	0.113	3.31	3.46	3.5	0.138	0.243	0.606	0.311
C18:1	41.5	42.08	0.35	41.69	41.57	42.11	0.429	0.246	0.650	0.908
C20:1	0.331	0.343	0.0136	0.329	0.33	0.352	0.0167	0.531	0.543	0.948
C18:2^2^	17.65^a^	16.88^b^	0.178	16.76^b^	17.26^ab^	17.77^a^	0.218	0.004	0.008	0.867
C20:2	0.187	0.182	0.0022	0.186	0.186	0.181	0.0027	0.115	0.345	0.798
C20:3	0.113	0.109	0.0028	0.113	0.109	0.11	0.0034	0.306	0.716	0.786
C20:4	1.81	1.62	0.066	1.64	1.76	1.74	0.081	0.053	0.559	0.800
C18:3	0.613	0.644	0.0226	0.58	0.642	0.663	0.0277	0.335	0.099	0.918
C20:5	0.217	0.205	0.0128	0.206	0.212	0.215	0.0156	0.526	0.915	0.969
C22:6	0.244	0.233	0.0124	0.233	0.236	0.248	0.0152	0.535	0.770	0.997
SFA^2^	33.83	34.38	0.227	34.95^a^	34.24^a^	33.11^b^	0.278	0.094	<0.001	0.891
MUFA	44.79	46.3	0.325	45.33	45.36	45.96	0.398	0.385	0.460	0.995
n-6 PUFA^2^	19.75^a^	18.79^b^	0.203	18.70^b^	19.31^ab^	19.80^a^	0.249	0.002	0.012	0.861
n-3 PUFA	1.07	1.08	0.029	1.02	1.09	1.13	0.036	0.826	0.102	0.887
Total PUFA^2^	20.83^a^	19.87^b^	0.208	19.72^b^	20.40^ab^	20.93^a^	0.255	0.002	0.007	0.846
PUFA/SFA^2^	0.607^a^	0.589^b^	0.0069	0.565^c^	0.596^b^	0.632^a^	0.0084	<0.001	<0.001	0.816
n-6/n-3 PUFA	18.62	17.7	0.507	18.54	18.2	17.74	0.621	0.211	0.660	0.956

Means within each column series with no superscript letters are not significantly different (*P* > 0.05). Values are means of 8 replicates per treatment combination with 4 eggs per replicate.

1 Saturated fatty acids (SFA) = C14:0 + C15:0 + C16:0 + C17:0 + C18:0 + C20:0; monounsaturated fatty acids (MUFA) = C16:1 + C18:1 + C20:1; n-6 polyunsaturated fatty acids (n-6 PUFA) = C18:2 + C20:2 + C20:3 + C20:4; n-3 PUFA = C18:3 + C20:5 + C22:6; and total PUFA = *n*-6 PUFA + *n*-3 PUFA.

2 There was a significant linear effect of dietary GAA levels (*P* < 0.001).

### Expression of lipid metabolism-related genes

**Fig 2:**
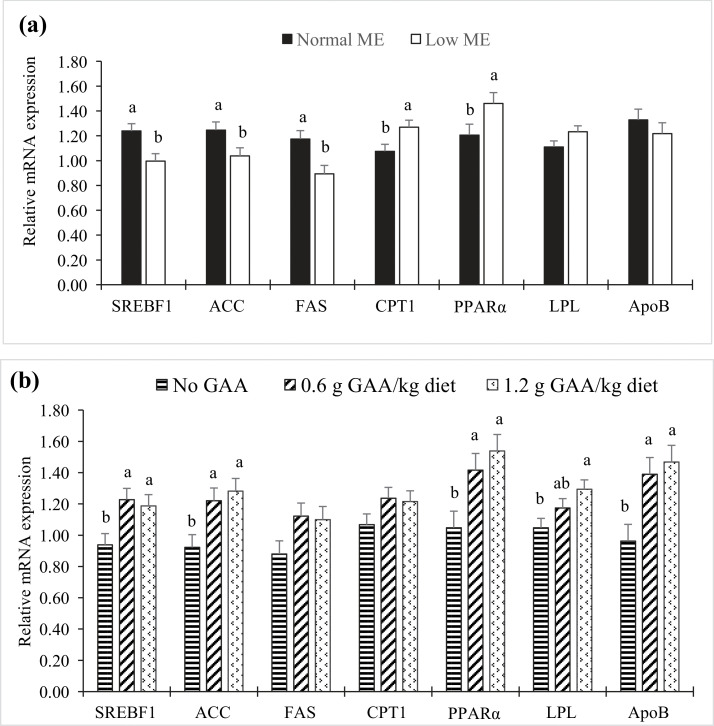
Bar charts of hepatic mRNA expression levels of [sterol regulatory element binding transcription factor 1 (SREBF1), acetyl‐coenzyme A carboxylase (ACC), fatty acid synthase (FAS), carnitine palmitoyltransferase 1 (CPT1), peroxisome proliferator activated receptor alpha (PPARα), lipoprotein lipase (LPL), and apolipoprotein B (ApoB)] in laying hens (at 64 weeks of age) according to the metabolizable energy (ME) content (a) and guanidinoacetic acid (GAA) supplementation (b) in the diets of laying hens. According to Tukey's test, mean values within the same histogram with no common superscript differ significantly (*P* < 0.05). Values are means of 8 replicates per treatment combination with 2 hens per replicate. No significant interaction effects were observed between ME and GAA supplementation in terms of lipid metabolism gene expression levels (*P* > 0.05). Dietary GAA levels had significant linear effects on mRNA expression levels of SREBF1, ACC, PPARα, LPL, and ApoB (*P* < 0.01).

Fig. 2 presents the expression patterns of hepatic genes involved in lipid metabolism. No significant interaction between ME and GAA supplementation was observed (*P* > 0.05). However, reducing dietary ME resulted in significant downregulation of SREBF1, ACC, and FAS, and upregulation of PPARα and CPT1 (*P* < 0.05). Increasing GAA levels induced a significant linear increase in SREBF1, ACC, PPARα, LPL, and ApoB expression (*P* < 0.05), but had no significant effects on FAS and CPT1 mRNA levels (*P* > 0.05).

### Intestinal morphology

**Fig. 3:**
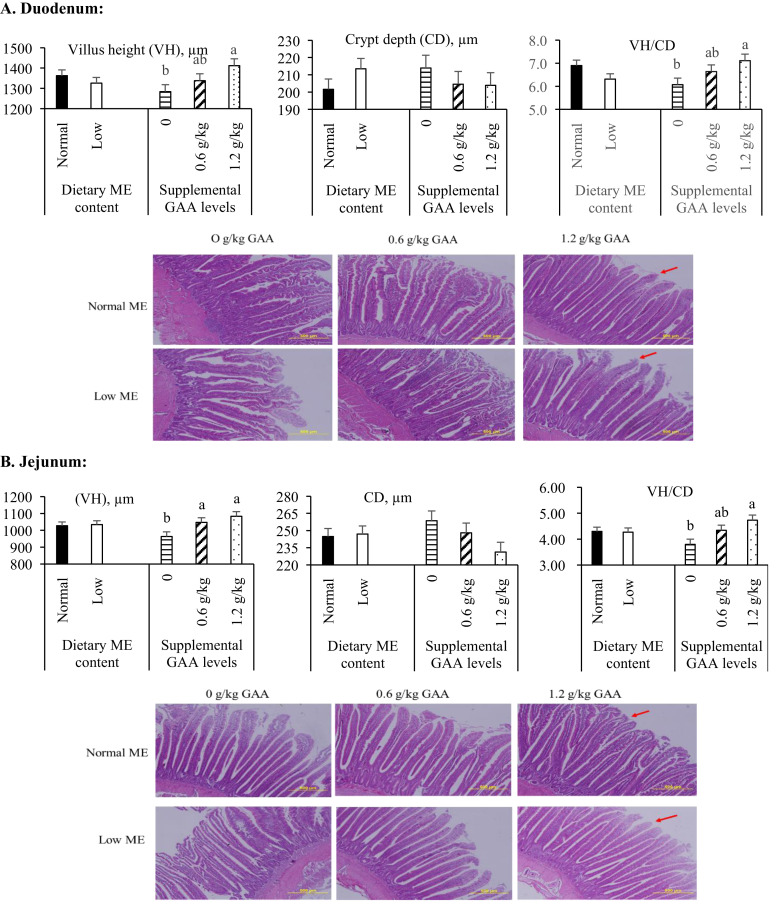
The morphological quantitative parameters and histological features (based on hematoxylin and eosin staining) of the mucosa of duodenum (A) and jejunum (B) in aged laying hens (64 weeks of age) according to metabolizable energy (ME) content and guanidinoacetic acid (GAA) supplementation in the diets. According to Tukey's test, mean values within the same histogram with no common superscript differ significantly (*P* < 0.05). No significant interaction effects were observed between ME and GAA supplementation in terms of morphological (*P* > 0.05). For histological observation, images at a lower magnification (100 ×) are provided. Red arrows highlight well-developed, elongated villi indicative of enhanced mucosal structure in comparison to other treatment groups.

Fig. 3 displays the histological features of the duodenum and jejunum. No significant interaction between ME and GAA was detected for morphological traits (*P* > 0.05). However, dietary GAA supplementation resulted in a linear increase in VH and VH/CD ratio in both intestinal segments (*P* < 0.05). Specifically, 1.2 g/kg GAA improved these parameters in the duodenum, while both 0.6 and 1.2 g/kg levels improved them in the jejunum. Histological observations confirmed these findings, with hens receiving 1.2 g/kg GAA exhibiting longer, denser villi, while non-supplemented groups showed shorter and more irregular villi.

### Nutrient digestibility

Table 8 summarizes the effects of dietary ME and GAA supplementation on nutrient digestibility. A significant interaction was observed for the AID of protein and AMEn values (*P* < 0.05), indicating that GAA enhanced these parameters under low-ME conditions. Compared to the normal-ME diet, the low-ME diet significantly reduced the AID of dry matter, energy, and AMEn (*P* < 0.05). Increasing dietary GAA levels led to linear and quadratic increases (*P* < 0.05) in protein digestibility, as well as a linear increase in AMEn values (*P* < 0.05). However, GAA did not significantly affect the AID of dry matter, fat, energy, or ash (*P* > 0.05).

**Table 8:** Apparent ileal digestibility of nutrients and AMEn (at 64 weeks of age) according to the metabolizable energy (ME) content and guanidinoacetic acid (GAA) supplementation in the diets of laying hens.

	GAA level (g/kg diet)	Dry matter	Crude protein	Crude fat	Ash	Energy	AMEn^1^
ME level		%					Kcal/kg
Normal	0	71.75	67.23^a^	80.03	48.84	71.89	2,666^a^
Normal	0.6	73.17	67.74^a^	80.11	50.32	72.76	2,671^a^
Normal	1.2	72.29	67.46^a^	80.26	49.96	73.93	2,679^a^
Low	0	70.06	65.10^b^	78.70	47.76	69.85	2,529^c^
Low	0.6	71.69	68.35^a^	79.33	49.74	71.04	2,575^b^
Low	1.2	71.18	67.85^a^	79.80	50.89	72.45	2,590^b^
SEM		0.812	0.502	0.692	1.120	1.066	10.0
Main effect							
ME							
Normal		72.40^a^	67.48	80.13	49.71	72.86^a^	2,672^a^
Low		70.98^b^	67.10	79.28	49.47	71.11^b^	2,565^b^
SEM		0.469	0.290	0.399	0.647	0.615	5.8
GAA level (g/kg diet)							
0		70.90	66.16^b^	79.37	48.30	70.87	2,598^b^
0.6		72.43	68.04^a^	79.72	50.03	71.90	2,623^a^
1.2		71.74	67.65^a^	80.03	50.43	73.19	2,634^a^
SEM		0.574	0.355	0.489	0.792	0.754	7.1
*P*-value							
ME level		0.037	0.382	0.140	0.783	0.050	<0.001
GAA level		0.183	0.001	0.624	0.144	0.108	0.002
ME × GAA		0.935	0.015	0.825	0.646	0.963	0.048
Effect of GAA level							
Linear		0.316	0.023	0.138	0.065	0.001	0.001
Quadratic		0.100	0.049	0.930	0.503	0.569	0.410

Means within each column series with no superscript letters are not significantly different (*P* > 0.05). Values are means of 8 replicates per treatment combination with 2 hens per replicate.

## Discussion

The present study investigated the effects of supplementing graded levels of GAA in laying hen diets with two different levels of ME on productive, metabolic, and nutritional responses. A reduction of 100 kcal/kg in dietary ME resulted in increased feed consumption, leading to a significantly higher FCR. In the unsupplemented groups, feed intake increased from 106.8 to 109.9 g/hen/day following ME reduction, while the overall mean feed intake across all treatments increased from 106.8 to 108.7 g/hen/day. These findings are consistent with previous research reporting approximately a 1 % increase in feed intake for every 33 kcal/kg reduction in dietary ME (Grobas et al., 1999; Wu et al., 2005). The study also found that dietary ME levels did not significantly affect laying rate, egg weight, or egg mass. These results align with earlier studies that similarly reported no significant effects of dietary ME content on egg production or egg weight in laying hens (Jalal et al., 2006; Gunawardana et al., 2009). It is well-established that dietary protein content exerts a greater influence on egg weight than dietary energy (Leeson and Summers, 2008). In the present study, all diets were formulated to contain identical levels of crude protein and amino acids, thereby ensuring that each group received adequate amounts of essential amino acids required to support egg size in accordance with the nutritional requirements of commercial laying hens. This dietary consistency likely explains the absence of an effect on egg weight observed in this experiment.

The current study demonstrated significant interactions between dietary ME content and GAA supplementation in relation to laying rate, egg mass, and FCR. Notably, the addition of GAA to low-ME diets, particularly at a level of 1.2 g/kg, significantly improved these performance metrics. In contrast, no substantial improvements were observed when GAA was added to normal-ME diets, suggesting that the efficacy of GAA may be modulated by the energy density of the diet. These findings indicate that the benefits of GAA supplementation are more pronounced under conditions of energy restriction. The positive effects of GAA on productive performance are likely attributable to its function as a direct precursor to creatine, a compound critical for cellular energy metabolism and involved in numerous physiological processes, including those associated with reproduction (Khajali et al., 2020). However, previous studies on GAA supplementation in laying poultry have reported inconsistent results. Some have documented improved laying performance following GAA inclusion (Raei et al., 2020; Salah et al., 2020; Azizollahi et al., 2024), whereas others have observed no significant benefits (Khakran et al., 2018; Pimenta et al., 2023). The inconsistencies observed across studies may be attributed to differences in the physiological status of the birds, dietary energy levels, and nutrient composition. In the present study, laying hens in the post-peak production phase (52 to 64 weeks of age) were fed diets containing either 2,700 or 2,600 kcal/kg AMEn, both formulated to provide equivalent protein and amino acid levels. Variations in these factors, along with differences in GAA dosage and supplementation duration, likely contributed to the inconsistent findings reported in the literature.

The 8 % reduction in FCR observed with low-ME + GAA supplementation indicates improved feed efficiency, which is a key determinant of both productivity and economic performance. This enhancement is likely attributable to increased metabolic efficiency resulting from greater creatine availability, which supports muscle function and energy production, thereby optimizing nutrient utilization and reproductive performance (Khajali et al., 2020). The 100 % increase in serum creatine reported in the present study (Table 6) further supports the association between GAA supplementation and enhanced energy metabolism, ultimately contributing to a higher laying rate and increased egg mass (Azizollahi et al., 2024).

In terms of egg quality, the results presented in Table 5 indicate that dietary ME content had no significant effect on either internal or external egg quality parameters. However, GAA supplementation, particularly at the highest level (1.2 g/kg), was associated with improved internal egg quality, as evidenced by a significant increase in Haugh unit values. This finding is consistent with previous research suggesting the beneficial effect of GAA on albumen quality (Salah et al., 2020). Given that the Haugh unit is a key indicator of egg freshness and overall quality (Eisen et al., 1962), dietary inclusion of GAA at 1.2 g/kg may serve as an effective strategy for enhancing internal egg quality, particularly in aging flocks. Additionally, GAA may contribute indirectly to improved egg quality through its known "sparing effect" on methionine and arginine. Dinesh et al. (2018) reported that both GAA and creatine can spare dietary methionine. Similarly, Reda et al. (2020) found that DL-methionine supplementation at 1.5 g/kg increased albumen percentage, while higher inclusion (3.5 g/kg) enhanced Haugh unit and shell thickness in quail breeders. This improvement in Haugh unit could be linked to GAA's role in supporting protein synthesis and energy metabolism. As GAA contributes to creatine synthesis, which in turn supports cellular energy metabolism (Ostojic, 2015), it may promote better protein synthesis within the albumen. The higher Haugh unit could therefore reflect enhanced albumen structure, which supports better overall egg quality (Vlčková et al., 2019). Additionally, the reduction in oxidative stress through GAA supplementation (as discussed later) might further contribute to the preservation of albumen quality.

As laying hens approach the later stages of their production cycle, eggshell quality typically declines. In the current study, a trend toward increased eggshell strength was observed with higher levels of dietary GAA, potentially due to its sparing effect on arginine, which plays a role in calcium absorption and metabolism (Yaman et al., 2016). This is supported by earlier studies showing that dietary arginine supplementation can improve eggshell thickness and promote calcium deposition (Lieboldt et al., 2016; Sahin et al., 2018). Oxidative stress is known to negatively affect eggshell quality, as free radicals and reactive oxygen species (ROS) can damage cellular components, including those involved in eggshell formation (Ding et al., 2022). GAA's antioxidant properties (Zhao et al., 2021) may help mitigate this oxidative stress, thus preserving eggshell integrity. Increased TAC in response to GAA supplementation, as observed in Table 6, could contribute to a reduction in oxidative damage, supporting healthier eggshell formation.

The study found that reducing dietary energy content did not significantly affect most measured blood parameters; however, it did lead to a significant increase in uric acid concentration. Previous research has demonstrated that a decrease in dietary energy prompts the body to metabolize amino acids, resulting in increased uric acid synthesis in birds (Hu et al., 2021). Supplementation with GAA during the post-peak phase of production in laying hens led to significantly elevated levels of serum creatine and creatinine. Similar findings have been reported in broiler chickens (Córdova-Noboa et al., 2018) and laying Japanese quails (Raei et al., 2020). It is essential to continuously replenish creatine, as a portion undergoes irreversible conversion to creatinine, which is excreted through urine (Wyss and Kaddurah-Daouk, 2000). GAA supplementation also resulted in significantly increased NO levels, which possess vasodilatory properties that enhance blood flow and oxygen delivery to the tissues and organs of poultry. These properties have been shown to positively impact the overall health and performance of poultry by improving nutrient uptake and metabolism (Dao and Swick, 2021; Miri et al., 2022). Furthermore, the study found that GAA supplementation significantly increased TAC and reduced MDA concentration, indicating a favorable effect on antioxidant capacity and lipid peroxidation. MDA is a well-established marker of oxidative stress, and its reduction suggests decreased oxidative damage (Ren et al., 2016). The DPPH radical scavenging activity is also an important indicator of a food's antioxidant capacity (Zhang et al., 2014; El-Tarabany and Ahmed-Farid, 2021). The findings revealed that egg yolks from hens that received GAA supplementation exhibited significantly lower TBARS values and higher DPPH activity, suggesting that dietary GAA enhances antioxidant defense mechanisms against oxidative stress in birds.

The study also determined that the optimal dosage of GAA for achieving maximum TAC and DPPH activity was 0.6 g/kg of diet. GAA may play a dual role, acting either as a pro-oxidant or an antioxidant depending on physiological conditions. It may directly function as a pro-oxidant by generating the superoxide anion (Hiramatsu, 2003), but it can also indirectly contribute to antioxidant capacity through its metabolites, arginine and creatine (Zhao et al., 2021). However, if GAA accumulates excessively or is metabolized improperly, it can increase oxidative stress (Ostojic, 2015). These findings suggest that, although GAA supplementation may improve the productivity and health of laying hens, appropriate dosing and continuous monitoring are essential to avoid potential adverse effects.

The primary factor influencing the fatty acid profile of egg yolks in avian species is the dietary fatty acid composition (Ruan et al., 2018). Our study found that a normal-ME diet increases both the PUFA content and the PUFA/SFA ratio in egg yolks. Our findings also indicate that dietary supplementation with GAA, particularly at a dosage of 1.2 g/kg, positively affects the fatty acid composition of egg yolks. Specifically, GAA supplementation significantly reduced SFA levels while increasing PUFA, particularly linoleic acid and arachidonic acid. These alterations suggest potential health benefits for consumers of eggs from hens fed GAA-supplemented diets. The PUFA/SFA ratio, an important indicator of lipid quality, also increased with higher concentrations of dietary GAA. The observed increases in specific fatty acids such as C18:2n-6 and C20:4n-6 in egg yolks following GAA supplementation may be due to GAA's role in enhancing the transport and deposition of these fatty acids into the yolk (Azizollahi et al., 2024). Another plausible explanation lies in the antioxidant properties of GAA (Amiri et al., 2019; Zhao et al., 2021), which may mitigate PUFA oxidation in tissues, including egg yolks, thereby preserving higher PUFA levels. Additionally, GAA's arginine-sparing effect may contribute to this improved fatty acid profile, as it enhances arginine availability. As shown in the present study (Fig. 2), supplementation with GAA significantly enhances the activity of including ACC, FAS, and PPARα, which are integral to the regulation of fatty acid biosynthesis, thereby increasing the synthesis of PUFA in the egg yolk. Specifically, ACC and FAS catalyze the formation of unsaturated fatty acids (Zhang et al., 2011), while PPARα facilitates the metabolic processing and incorporation of PUFA (Royan et al., 2011), contributing to their elevated accumulation in the yolk. Moreover, the upregulation of LPL and ApoB by GAA in the current study further supports enhanced fatty acid uptake and transport, resulting in more efficient PUFA deposition into the yolk and a corresponding increase in the PUFA/SFA ratio. Nonetheless, further research is necessary to fully elucidate the mechanisms by which elevated levels of GAA influence the fatty acid composition of egg yolks.

In birds, de novo lipogenesis is particularly active relative to other metabolic processes such as oxidation and lipolysis, and this pathway is especially prominent in the liver of sexually mature birds (Ruan et al., 2015). The present study evaluated the effects of low-ME diets and GAA supplementation on the expression of genes related to lipogenesis and lipolysis in laying hens. The results indicated that low-ME diets suppressed lipogenic activity while promoting lipolytic pathways, as evidenced by significant alterations in gene expression. Specifically, there was a marked downregulation of lipogenesis-related genes (SREBF1, FAS, and ACC), coupled with an upregulation of lipolysis-associated genes (PPARα and CPT1). These transcriptional changes suggest a metabolic shift toward decreased fatty acid synthesis and increased reliance on fatty acid oxidation for energy production. This finding aligns with previous research showing that energy-rich diets upregulate SREBF1 and ACC, and downregulate CPT1 in the livers of broiler chickens (Ge et al., 2019).

Additionally, GAA supplementation was found to significantly upregulate SREBF1 and ACC, indicating increased triacylglycerol synthesis and a heightened capacity for very low-density lipoprotein (VLDL) secretion in laying hens. This suggests that GAA enhances the synthesis and mobilization of fat from the liver to the egg yolk, potentially contributing to improved egg quality. Interestingly, despite these molecular changes, serum triglyceride and cholesterol levels remained unaffected, possibly due to homeostatic regulatory mechanisms that maintain lipid balance. Moreover, GAA supplementation upregulated the hepatic expression of LPL and ApoB. LPL is crucial for the hydrolysis of triglycerides (Sato et al., 2010), while ApoB is a key structural component of VLDL-yolk particles responsible for lipid transport to the ovary for yolk deposition (Zhou et al., 2014). These gene expression patterns suggest that GAA can modulate lipid deposition and distribution in laying hens. Supporting this, a recent study found that dietary GAA at 400, 600, and 800 mg/kg increased the relative expression of fatty acid transport protein 1 and LPL in the breast muscle and liver of ducks (Wu et al., 2024). The mechanism by which GAA affects hepatic gene expression may involve its conversion to creatine or its arginine-sparing function. Creatine pyruvate, a compound consisting of 40 % pyruvic acid and 60 % pharmaceutical-grade creatine, has been shown to increase CPT1 and PPARα expression in broiler livers, thereby promoting fatty acid oxidation (Chen et al., 2011). Given the elevated rate of fat metabolism in laying hens associated with egg production (van Eck et al., 2023), it is likely that genes involved in lipid synthesis, oxidation, and transport are activated simultaneously to meet the metabolic demands of the ovary and other tissues. Thus, GAA supplementation may stimulate both lipogenic and lipolytic gene expression, thereby promoting increased lipid turnover. This metabolic adaptation may contribute to enhanced egg production and improved egg mass in laying hens.

The intestinal morphology of avian species plays a crucial role in maintaining overall health and optimizing performance. A well-developed and healthy intestinal structure is essential for efficient nutrient absorption, robust immune function, and sustained growth (Wu et al., 2018; Bean-Hodgins et al., 2022). Consequently, identifying strategies to enhance intestinal morphology is of significant interest to the poultry industry. The present study investigated the potential positive effects of GAA on the development of intestinal mucosa in aged laying hens fed diets with two different ME levels. The findings indicated that dietary ME content had no significant effect on the morphological features of the duodenal and jejunal mucosa. These results are consistent with previous studies (Attia et al., 2021; Wickramasuriya et al., 2023), which similarly reported no notable alterations in gut morphology in broiler chickens when dietary ME was reduced by 100 or 150 kcal/kg, respectively.

In contrast, GAA supplementation was found to exert beneficial effects on the morphological parameters of the intestinal mucosa in both the duodenum and jejunum. Notably, the highest inclusion level of GAA (1.2 g/kg) produced the most favorable outcomes, particularly in the duodenum. To the best of our knowledge, limited prior research has explored the effects of GAA on gastrointestinal morphology in laying hens. However, studies in broilers have reported improvements in intestinal structure following GAA supplementation under various environmental conditions, including thermoneutral (Ahmadipour et al., 2018), heat stress (Amiri et al., 2019), and cold stress environments (Miri et al., 2022). Although the precise mechanism underlying these improvements remains to be fully elucidated, the observed enhancements in gut morphology may be linked to the beneficial effects of arginine on intestinal health. Arginine is known to stimulate the upregulation of genes in the mechanistic target of rapamycin (mTOR) signaling pathway, which plays a central role in cellular growth and protein synthesis (Yuan et al., 2015). By promoting protein synthesis, arginine may facilitate the production of essential structural and functional proteins that maintain intestinal integrity. Additionally, arginine has been shown to reduce protein degradation, thereby preserving critical proteins required for intestinal maintenance and function (Gao et al., 2018). This dual effect of increased synthesis and reduced degradation may underlie the positive impact of GAA on intestinal morphology, given its arginine-sparing properties. Creatine boosts ATP production in the intestine, supporting enterocyte function and intestinal integrity (Hall et al., 2020). Therefore, GAA could enhance energy availability, promoting cell repair and growth, which improves intestinal morphology. Additionally, GAA increases nitric oxide (NO) production, enhancing blood flow and nutrient delivery to the intestines, supporting epithelium growth and tissue regeneration (Dao and Swick, 2021).

The results presented in Table 8 indicate that a low-ME diet had a detrimental effect on AMEn and the AID of energy and dry matter in laying hens. In contrast, the AID of fat, protein, and ash was not significantly influenced by dietary ME content. The observed decline in AMEn, dropping from 2672 to 2565 kcal/kg when dietary ME was reduced by 100 kcal/kg, was anticipated. Additionally, the study found significant interaction effects between dietary ME level and GAA supplementation on the AID of protein and AMEn. These findings suggest that GAA supplementation can enhance protein utilization and AMEn under low-ME conditions, though it may not confer additional benefits in diets with adequate energy levels. Similar findings have been reported in laying Japanese quails, where GAA supplementation was shown to increase the digestibility of various nutrients (Raei et al., 2020). The positive influence of GAA is likely mediated through its role in improving creatine synthesis and enhancing energy metabolism in intestinal epithelial cells (Wallimann et al., 2011). Therefore, dietary inclusion of GAA may support improved absorption and utilization of protein and energy, particularly under energy-deficient dietary conditions.

## Conclusions

In conclusion, reducing dietary ME content by 100 kcal/kg adversely affected feed efficiency, lipid metabolism, and nutrient digestibility in laying hens, although no significant impacts were observed on egg quality or antioxidant status. The study also demonstrated that GAA supplementation improved productive performance, protein digestibility, and AMEn in low-ME diets. Moreover, GAA exerted beneficial effects on egg quality, yolk fatty acid composition, and antioxidant status regardless of dietary ME content. In addition to these physiological benefits, GAA was found to upregulate the expression of genes involved in lipid synthesis, lipolysis, and fatty acid transport in the liver, indicating enhanced lipid turnover. Based on the findings of this study, a dietary inclusion level of 0.6 g/kg GAA is recommended for laying hens during the later stages of production. However, further research is warranted to elucidate the underlying mechanisms, as well as to determine the optimal dosage and supplementation duration across different phases of the laying cycle.

## Declaration of competing interest

The authors declare that the study was carried out without any financial or commercial relationships that could be construed as a potential source of conflict of interest.
